# Prevalence of extended-spectrum *β*-lactamase-producing *Enterobacterales* in retail sheep meat from Zagazig city, Egypt

**DOI:** 10.1186/s12917-022-03294-5

**Published:** 2022-05-20

**Authors:** H. M. Abdallah, N. Al Naiemi, Ibrahim Elsohaby, Abdallah F. A. Mahmoud, Gamal A. Salem, C. M. J. E. Vandenbroucke-Grauls

**Affiliations:** 1 Department of Microbiology, Faculty of Veterinary Medicine, Zagazig University, Zagazig, Egypt; 2 Medical Microbiology and Infection Control, VU University Medical Center, Amsterdam, The Netherlands; 3 Department of Infectious Diseases and Public Health, Jockey Club of Veterinary Medicine and Life Sciences, City University of Hong Kong, Kowloon, Hong Kong, China; 4 Department of Animal Medicine, Division of Infectious Diseases, Faculty of Veterinary Medicine, Zagazig University, Zagazig, Egypt; 5 Food Control Department, Faculty of Veterinary Medicine, Zagazig University, Zagazig, Egypt; 6 Department of Pharmacology, Faculty of Veterinary Medicine, Zagazig University, Zagazig, Egypt

**Keywords:** ESBL, Antimicrobial, Egypt, Resistance

## Abstract

**Background:**

The goal of this study was to investigate the prevalence of extended-spectrum *β*-lactamase production in *Enterobacterales* isolated from retail sheep meat in Zagazig, Egypt.

**Methods:**

One hundred random samples of sheep meat were collected from different retail butcher shops (*n* = 5) in the city of Zagazig, Egypt. Bacterial isolates were identified by MALDI-TOF MS and screened for antibiotic susceptibility by disk diffusion; further genotypic characterization of *β*-lactamase-encoding genes was performed with Real-Time PCR. *E. coli* strains were phylotyped with the Clermont triplex PCR method.

**Results:**

Of the total of 101 bacterial isolates recovered from retail sheep meat samples, 93 were *E. coli*, six were *Enterobacter cloacae* and two were *Proteus mirabilis*. As many as 17% of these 100 samples showed ESBL phenotypes, all were *E. coli*. The *bla*_CTX-M_ genes were detected in seven isolates (six were *bla*_CTX-M-15_ and one was *bla*_CTX-M-14_), three isolates harboured *bla*_TEM_ (all were bla_TEM-one_), and two carried genes of the *bla*_SHV_ family (both were *bla*_SHV-12_). Eight *E. coli* isolates expressed ESBL phenotype but no *bla*_TEM_, *bla*_SHV_ or *bla*_CTX-M_ genes were detected by PCR. ESBL- positive *E. coli* isolates were nearly equally distributed over the commensal groups A/B1 and the virulent group D.

**Conclusion:**

Nearly one in five sheep meat samples was contaminated with ESBL-*E. coli.* This further corroborates the potential role played by contaminated meat in the increasing resistance rates that have been reported worldwide.

## Background

Since the breakthrough discovery of penicillin in the 1928s, *β*-lactam antibiotics have saved countless lives, but it didn't take long for *β*-lactam-resistant bacteria to be identified [1]. The production of *β*-lactamases by enzymatic hydrolysis of the *β*-lactam ring is the primary contributor to *β*-lactam resistance [2]. Extended-spectrum beta-lactamases (ESBLs) are of particular concern among these enzymes because they inactivate extended-spectrum cephalosporins [3]. These enzymes can be produced by a wide range of bacteria, including *Enterobacterales* and non-fermenting bacteria [3–5]. *Escherichia coli* is the most common ESBL-producing species, and it frequently causes urinary tract infections, pneumonia, and even sepsis in humans [6].

According to recent studies, animals may serve as a reservoir for these ESBL-producing *Enterobacterales* [7–9]. The possibility that these antimicrobial-resistant *Enterobacterales* of animal origin are transmitted to humans via the food chain has been considered [10]. Furthermore, evidence of a link between antimicrobial use in food-producing animals and human resistance has been reported [11].

The contamination of raw meats with ESBL-producing *Enterobacterales* (ESBL-E) is a growing problem because they play a potential role in the spread of ESBL genes to humans via food chains [12]. ESBL-E contamination of raw retail meats has been detected in studies all over the world [13–16]. In Egypt, the national antimicrobial stewardship program has been established but there are no strict laws to enforce its implementation [17, 18]. Antimicrobials such as tetracycline, quinolones, and beta lactams are still used in Egypt for animal feed growth promotion and by veterinarians to treat and prevent zoonotic diseases [19]. There is a scarcity of data on ESBL-producing bacteria in Egyptian food animals. In a previous study, we discovered that ESBL-E was present in 63% of Egyptian retail chicken meat samples [9]. Because of the high ESBL-E contamination rate, the aim of this current study was to determine the prevalence of ESBL-E in retail sheep meat from Zagazig, Egypt.

## Results

**Fig. 1:**
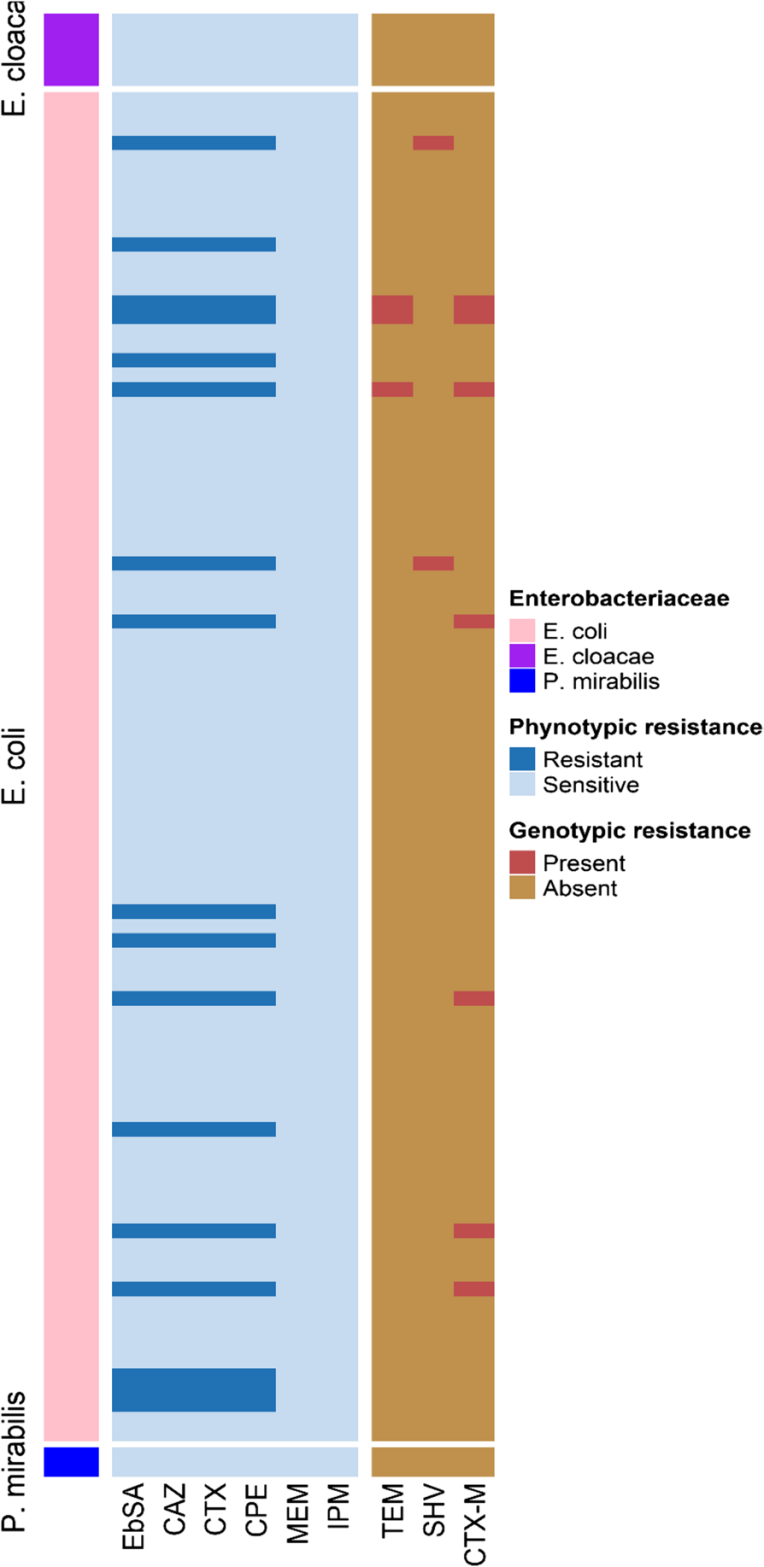
Overview of antimicrobial resistance pattern. Antimicrobial resistant genes of all *Enterobacterales* strains isolated from 100 retail sheep meat collected from Zagazig city, Egypt

Out of 100 retail sheep meat samples, 101 enterobacterial isolates were recovered, 93 were *E. coli*, six were *Enterobacter cloacae* and two were *Proteus mirabilis*. Putative ESBL-E isolates were identified in 17 samples (Table 1). All isolates with ESBL phenotype belonged to *E. coli*. All isolates were susceptible to meropenem and imipenem **(**Fig. 1**)**.

**Table 1:** *Enterobacterales* strains isolated from 100 retail sheep meat samples collected from Zagazig, Egypt

Species	No. of isolates	No. of ESBL positive isolates (%)
*E. coli*	93	17 (18.3)
*Enterobacter cloacae*	6	0 (0)
*Proteus mirabilis*	2	0 (0)
Total	101	17 (16.8)

*bla*_CTX-M_ were identified in 41.18% (7/17) of the ESBL-producing *E. coli*, whereas *bla*_TEM_ and *bla*_SHV_ were detected in 17.65% (3/17) and 11.76% (2/17), respectively. Concomitant presence of *bla*_CTX-M_ and *bla*_TEM_ was detected in 3 isolates, 4 isolates expressed *bla*_CTX-M_ alone, and 2 harboured only *bla*_SHV_. Eight *E. coli* isolates expressed ESBL phenotype but no *bla*_TEM_, *bla*_SHV_ or *bla*_CTX-M_ genes were detected by PCR (Table 2).

**Table 2:** Characteristics of ESBL-producing *E. coli* strains isolated from 100 retail sheep meat collected from Zagazig, Egypt

	**Phylogenetic groups**				**Total**
	**A**	**B1**	**B2**	**D**	
**ESBL positive *E. coli***					
CTX-M-15	1	1	0	1	3
CTX-M-15 + TEM-one	0	0	0	3	3
CTX-M-14	0	0	0	1	1
SHV-12	2	0	0	0	2
No Enzymes	3	1	0	4	8
** Subtotal**	**6**	**2**	**0**	**9**	**17**
**ESBL Negative *E. coli***					
** Subtotal**	**27**	**46**	**1**	**2**	**76**
** Total**	**33**	**48**	**1**	**11**	**93**

Of the seven *bla*_CTX-M_ – positive *E. coli* isolates, six (85.7%) were *bla*_CTX-M-15_ positive, and one *bla*_CTX-M-14_. All the three TEM genes were *bla*_TEM-one_ while the two *bla*_SHV_-type ESBL genes were identified as *bla*_SHV-12_*.*

**Fig. 2:**
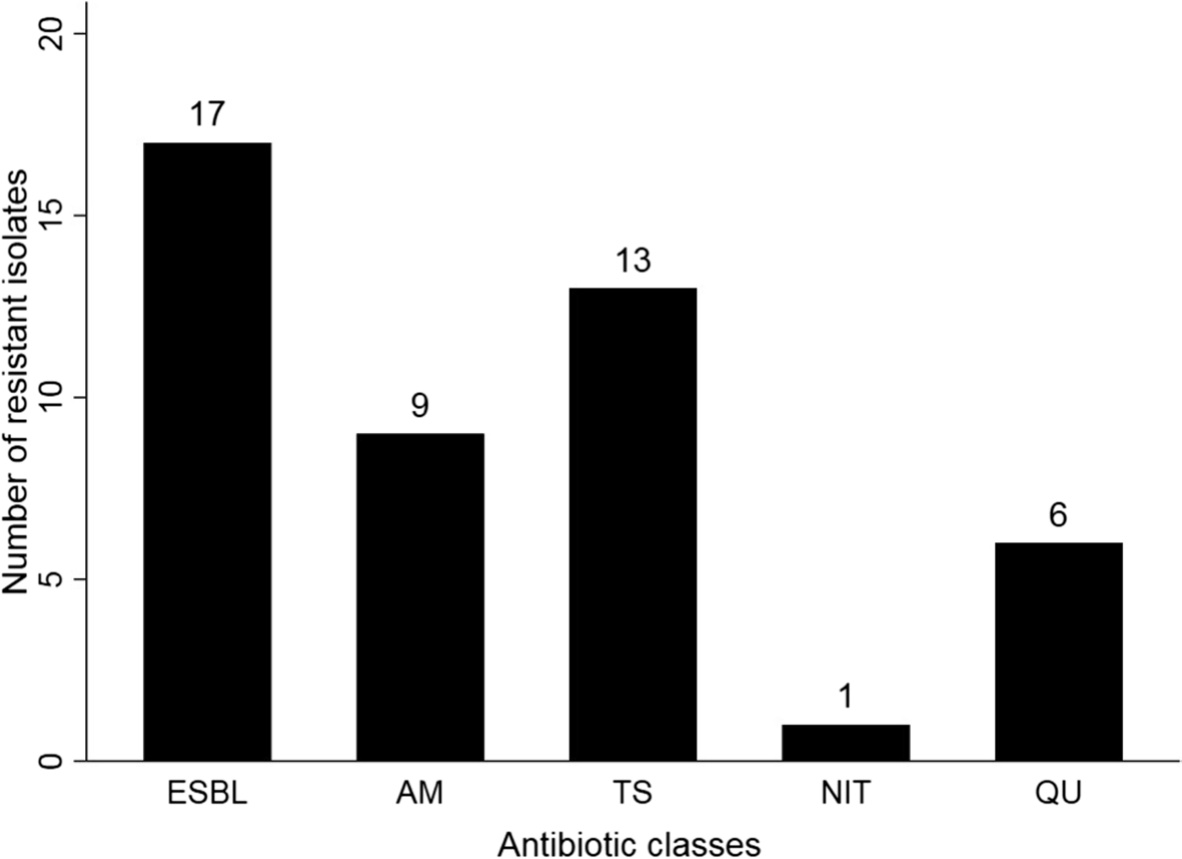
Antimicrobial resistance profile of *E. coli* strains isolated from 100 retail sheep meat collected from Zagazig, Egypt (ESBL, extended-spectrum beta-lactamases; AM, aminoglycosides; TS, trimethoprim/sulfamethoxazole; NIT, nitrofurantoin; QU, quinolones)

Disc-diffusion antimicrobial susceptibility testing revealed that of 17 ESBL-producing isolates, 13 (76.47%) were resistant to trimethoprim/sulfamethoxazole, 9 (52.94%) to aminoglycosides, 6 (35.29%) to quinolones, and only one to nitrofurantoin, while 5 (29.41%) were multidrug resistant (resistant to three or more antimicrobial classes) **(**Fig. 2**)**.

Phylogenetic grouping of 17 ESBL-positive *E. coli* isolates showed that six isolates belonged to group A, two to group B1, nine to group D, while no isolates belonged to group B2. The 76 ESBL-negative isolates were: 27 group A, 46 group B1, one group B2 and two group D (Table 2).

## Discussion

Extended-spectrum *β*-lactamase-producing bacteria are one of the fastest emerging resistance problems worldwide [15]. Livestock may be an important vehicle for the community-wide dissemination of ESBL-producing bacteria [7]. In Egypt, the role of food-producing animals has not been fully assessed; nothing is known about possible contamination of sheep meat with ESBL-producing *Enterobacterales* and their encoding genes. Our study showed that all retail meat samples were contaminated with *Enterobacterales*; of these over 90% was *E. coli.* The frequency of *E. coli* among contaminating *Enterobacterales* coincides with what has been described earlier in other studies [20–22].

This study revealed that nearly one in five *E. coli* isolates was ESBL positive, showing that also sheep meat may be a source of ESBL-producing strains for humans. Sheep meat, however, appeared less contaminated than chicken meat in Egypt. We have shown previously that in the same region in Egypt, over 65% of retail chicken meat samples were positive for ESBL-E [9]. Possibly, the difference in contamination rates between chicken and sheep meat owes to differences in the production system, which is more intensive in poultry industry than in the sheep rearing system [23–25]. In our study, the frequency of ESBL-producing *E. coli* was higher to that reported for sheep meat in Switzerland (8.6%) [26] and Portugal (5.5%) [27], while it was lower than the 60% reported in Iran [28], 63.8% detected in chicken meat in Tunisia [29], 27.5% found in ground beef samples in Algeria [30] and 23% identified among imported chicken meat in Gabon [31]. The difference in prevalence of ESBL-E between these countries could be attributed to poor antibiotic use regulations in Middle East unlike the restricted policy of antibiotic use adopted by EU countries [32].

In this study, various types of ESBL-encoding genes were identified including *bla*_CTX-15_, *bla*_CTX-14_, *bla*_TEM-one_ and *bla*_SHV-12_. Our results are similar to those of a previous report from Egypt, in which *bla*_CTX_, *bla*_TEM_ and *bla*_SHV_ were found in ESBL-producing *E. coli* recovered from meat and dairy farms [9, 33]. However, the occurrence of *β*-lactamase genes in our study is higher than in recent reports from Turkey [34], Switzerland [26], Portugal [27] and Japan [35]. Regarding the types of *bla*_CTX-M_ gene, our data showed that *bla*_CTX-M-15_ was the most frequent ESBL-type in our *E. coli* collection. This is consistent with our finding that *bla*_CTX-M-15_ was also the most frequent ESBL in *E. coli* from chicken meat in Egypt. In other countries, e.g. Switzerland and Portugal, *bla*_CTX-M-14_ appeared as the most prevalent gene in *E. coli* isolates from sheep meat [26, 27] while in Gabon, Tunisia and Algeria the *bla*_CTX-M-one_ was predominant in ESB-*E. coli* from meat samples [29–31]. Eight *E. coli* showed ESBL phenotype, but they were negative for screened ESBL genes, this could be attributed to production of unscreened minor ESBL genes as OXA-type beta lactamases.

In the present study, the ESBL-producing *E. coli* isolates showed high frequency of co-resistance to trimethoprim/sulfamethoxazole, aminoglycosides, quinolones and nitrofurantoin, which is similar to other reports on antimicrobial resistance of *E. coli* isolates recovered from retail meat in Egypt [9, 33], China [22], Turkey [36], and Italy [37]. This multi-resistance trait showed that nearly 40% (*n* = 5) of the isolates were multidrug resistant (MDR). Similarly, high levels of MDR isolates recovered from sheep meat [27], retail chicken meat [9], and beef meat [21] have been reported in Portugal, Egypt and Spain, respectively. The presence of a high level of MDR isolates could be related to the unrestricted usage of antibiotics in food animals and farms [25]. Phylogenetic grouping of ESBL positive *E. coli* revealed a uniform distribution of ESBL genes among virulent and avirulent phylogenetic groups, inconsistent with antibiotic resistance—virulence trade off hypothesis [38]. In addition, the distribution of phylogenetic groups may vary according to the geographic regions [39].

## Conclusions

Our findings highlight the possible role played by contaminated sheep meat as a source of antibiotic-resistant bacteria in Egypt. The high prevalence of ESBL-producing multidrug-resistant *Enterobacterales* detected in retail sheep meat, increases the concern regarding human exposure to superbugs. Thus, to tackle antibiotic resistance in the human–animal interface, proactive efforts should be taken to establish national action plans based on the One Health approach [40]***.***

## Methods

### Study area

**Fig. 3:**
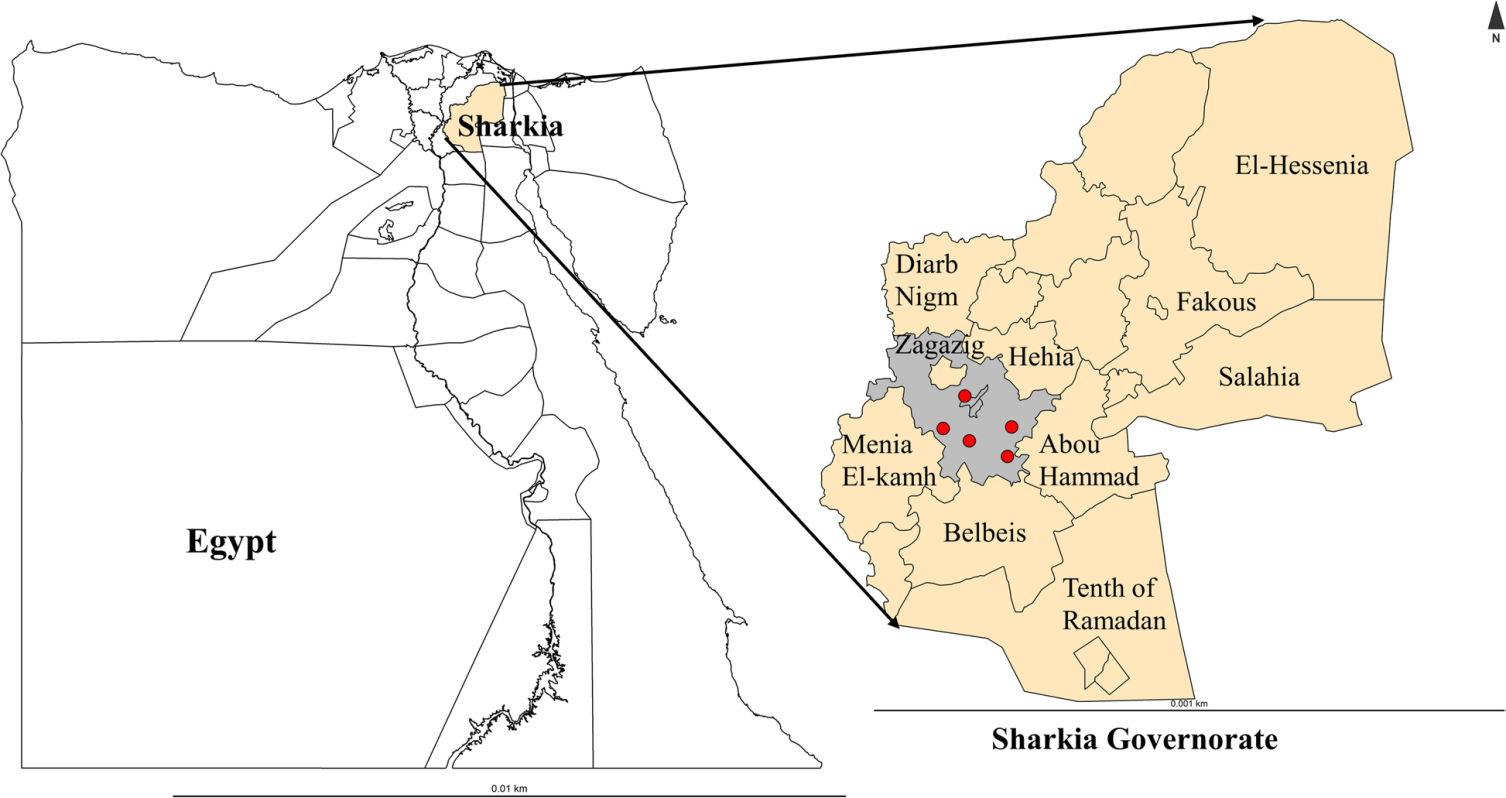
Map of Egypt showed the location of Zagazig city (grey colour) in Sharkia governorate and the location of the five retail butcher shops in Zagazig city (red dots). The map was created using R software (R Core Team, 2019; version 3.5.3) and “cartography” and “sf” packages

This study was performed in Zagazig city, which is the capital of Sharkia governorate, Egypt. Zagazig city is located in the northern part of Egypt at latitude 30°35′15″ N; longitude 31°30′07″ E and altitude 16 m above sea level (Fig. 3). Sharkia governorate considered the third populous governorate in Egypt, has a strong an agriculture industry and has also a high density of ruminants (cattle, sheep and goats) which are used mainly for meat production.

### Study design and sampling strategy

A cross-sectional study was performed from January 2013 to May 2013. The required number of sheep meat samples was determined using the formula for simple random sampling, with 10% expected prevalence, 5% absolute precision and 95% confidence interval. In total, 100 samples of sheep meat were collected from five retail butcher shops in Zagazig city. The shops were visited once bi-weekly. At each visit, two random meat samples were purchased from each shop, and immediately transported to the laboratory for culture.

### Isolation and identification of *Enterobacterales*

Sampling was performed by swabbing–based method [41]. Each swab was immersed in 5 mL of physiological saline solution (0.9%), mixed well by vortexing for 10 s, centrifuged at 3,500 × g for 15 min, most of the supernatant was decanted and 100 µL of the sediment was inoculated directly on selective EbSA-ESBL Screening Agar [42] for the characterization of extended-spectrum cephalosporin-resistant Gram-negative bacteria and on MacConkey agar for the isolation of the dominant bacteria. A pure bacterial colony was picked up from both culture plates for further identification by the automated Vitek® MS system (BioMérieux, Marcy l’Étoile, France).

### Phenotypic screening and confirmation of ESBL-E

Bacterial isolates were tested for antibiotic susceptibility by disk diffusion method on Mueller–Hinton agar using ceftazidime (30 μg), cefotaxime (30 μg), cefepime (30 μg), meropenem (10 μg), imipenem (10 μg), nitrofurantoin (100 μg), norfloxacin (10 μg), gentamicin (10 μg), and trimethoprim/sulfamethoxazole (1.25–23.75 µg) disks. Antibiotic inhibition zone diameters were evaluated in conformity with to CLSI–approved interpretive criteria [43]. Combination disks method was employed to confirm ESBL production, according to the guidelines of the Dutch Society of Medical Microbiology [44].

### Genotypic characterization of *β*-lactamase-encoding genes

ESBL phenotypes were tested for genes encoding *bla*_TEM_, *bla*_SHV_ and *bla*_CTX-M_ by real-time PCR using primers described before [45–47]. Subsequently, sequencing was performed with the Sanger ABI 3730 XL automated DNA sequencer (BaseClear, Leiden, The Netherlands), and analysis was performed with the Codon Code Aligner software (Version 5.0.2). The obtained nucleotide sequences were compared with described sequences available at the National Center for Biotechnology Information website (www.ncbi.nlm.nih.gov).

### *E. coli* phylotyping

Assignment of *E. coli* isolates to phylotypes (A, B1, B2 or D) was done based on the Clermont triplex PCR method targeting *chuA*, *yjaA* and the *TspE4.C2* DNA fragment [48].

### Data analysis

Information collected, and the antimicrobial resistance results were coded and entered into Microsoft Excel, and the descriptive statistical data analysis was performed using STATA version 15 for Windows (Stata Corp., USA). However, the heatmap was created using the R package “Complex-Heatmap” [49].

## Data Availability

The datasets generated and/or analysed during the current study are available in the [Figshare] repository, [https://figshare.com/articles/dataset/Raw_data_of_ESBL-E_in_retail_sheep_meat/19139714].

## Abbreviations

*E. coli*: *Escherichia coli*
PCR: Polymerase chain reaction
ESBLs: Extended-spectrum beta-lactamases
MDR: Multidrug resistant
CLSI: Clinical and laboratory standards institute
